# Kassiopeia: a database and web application for the analysis of mutually exclusive exomes of eukaryotes

**DOI:** 10.1186/1471-2164-15-115

**Published:** 2014-02-10

**Authors:** Klas Hatje, Martin Kollmar

**Affiliations:** 1 Group Systems Biology of Motor Proteins, Abteilung NMR basierte Strukturbiologie, Max-Planck-Institut für Biophysikalische Chemie, Am Fassberg 11, Göttingen D-37077, Germany

**Keywords:** Mutually exclusive splicing, Database, Web application, Drosophila

## Abstract

**Background:**

Alternative splicing is an important process in higher eukaryotes that allows obtaining several transcripts from one gene. A specific case of alternative splicing is mutually exclusive splicing, in which exactly one exon out of a cluster of neighbouring exons is spliced into the mature transcript. Recently, a new algorithm for the prediction of these exons has been developed based on the preconditions that the exons of the cluster have similar lengths, sequence homology, and conserved splice sites, and that they are translated in the same reading frame.

**Description:**

In this contribution we introduce Kassiopeia, a database and web application for the generation, storage, and presentation of genome-wide analyses of mutually exclusive exomes. Currently, Kassiopeia provides access to the mutually exclusive exomes of twelve *Drosophila* species, the thale cress *Arabidopsis thaliana*, the flatworm *Caenorhabditis elegans*, and human. Mutually exclusive spliced exons (MXEs) were predicted based on gene reconstructions from Scipio. Based on the standard prediction values, with which 83.5% of the annotated MXEs of *Drosophila melanogaster* were reconstructed, the exomes contain surprisingly more MXEs than previously supposed and identified. The user can search Kassiopeia using BLAST or browse the genes of each species optionally adjusting the parameters used for the prediction to reveal more divergent or only very similar exon candidates.

**Conclusions:**

We developed a pipeline to predict MXEs in the genomes of several model organisms and a web interface, Kassiopeia, for their visualization. For each gene Kassiopeia provides a comprehensive gene structure scheme, the sequences and predicted secondary structures of the MXEs, and, if available, further evidence for MXE candidates from cDNA/EST data, predictions of MXEs in homologous genes of closely related species, and RNA secondary structure predictions. Kassiopeia can be accessed at http://www.motorprotein.de/kassiopeia.

## Background

Alternative splicing is an important mechanism to increase and regulate the protein content of eukaryotic cells. There is evidence that about 95% of human multi-exon genes undergo alternative splicing [1]. One type of alternative splicing is mutually exclusive splicing, in which exactly one exon of a cluster of several neighbouring exons is spliced into the messenger RNA. The splicing of these mutually exclusive spliced exons (MXEs) is often highly regulated in a tissue-specific manner. In some of the best analysed genes, the *Drosophila DSCAM* (Down syndrome cell adhesion molecule), *Mhc* (muscle myosin heavy chain) and *14-3-3-ζ* genes, but also in human genes like *dynamin-1*[2], splicing of the MXEs is regulated by competing RNA secondary structures formed by conserved motifs within the introns and complementary acceptor sequences in either the 5’ or 3’ intron [3,4]. In humans, missense mutations in MXEs can lead to diseases [5,6].

Different approaches have been followed to identify alternatively spliced isoforms of genes. There are many genome-wide studies based on transcriptome sequencing (RNA-Seq), cDNA sequencing, and tiling microarrays (see for example [7-9]). The analysis of tandem mass spectra against genomic databases is also increasingly been used to identify alternatively spliced genes [10]. In contrast to these high-throughput experimental data methods, computer based *de novo* predictions of alternative splicing events are not well established yet. In one approach support vector machine classifiers have been built from gene features that have experimentally been shown to effect alternative splicing [11]. Other approaches used bayesian networks to predict NAGNAG tandem acceptor splice sites [12], genetic programming to classify cassette exons versus retained introns [13], and *ab initio* gene prediction methods [14]. Further, virtual genetic coding schemes combined with time series analyses have been used to predict alternatively spliced genes in *Caenorhabditis elegans*[15].

Recently, we introduced a new method to predict MXEs based on several preconditions to create biological meaningful transcripts [16]. We presumed that exons of a cluster of MXEs encode the same region and thus the same secondary structural elements of the resulting protein structure. Two prominent examples are the arthropod *Mhc*[17] and *DSCAM* genes [18]. The preconditions for MXEs are therefore similar length (sequence length should be fixed in regions forming α-helices and β-strands but slightly flexible in loop regions), conserved splice site patterns (only certain combinations of 5’- and 3’-splice sites are possible), the preservation of the reading frame, and sequence homology. These conditions have been implemented into an algorithm with which many new exon candidates were proposed as part of an analysis of the genome of *Drosophila melanogaster*[19].

In order to facilitate the production of datasets of mutually exclusive exomes and to provide a helpful interface for their analysis and presentation we have developed a web application, which we called Kassiopeia. We generated and integrated data for twelve *Drosophila* species, which are well known to contain many mutually exclusive spliced genes including the highly complex *DSCAM* gene [20], and for the plant *Arabidopsis thaliana* and the nematode *Caenorhabditis elegans*, for which reports about mutually exclusive spliced genes are rare. In addition, preliminary data of the human genome is included. Kassiopeia can be accessed at http://www.motorprotein.de/kassiopeia.

## Construction and content

### The database

The database management system is PostgreSQL. The table *proteins* is in the center of the database model with one record for each protein. Each *proteins* record contains the name of the dataset, the name of the protein, additional identifiers like the Genbank ID, NCBI gi and Flybase identifier, the genome target identifier/name, the genomic position of the locus containing the gene coding for the protein, and annotations. The annotations include the completeness of the Scipio gene structure reconstruction, and the presence of predicted MXE candidates and constitutive exons matching the criteria of MXEs. Each protein is linked to a gene, which is stored in the *genes* table. The *genes* table contains fields for the dataset name, the target identifier, the position of the gene locus, the name of the gene, additional identifiers like the Flybase ID and Genbank ID, and the presence of annotated MXEs, which were identified by comparing protein isoforms. Each *proteins* record is further linked to a table containing the corresponding gene structure reconstructed with Scipio [21], and, if appropriate, further tables containing EST data mappings, cross-species search results, and RNA secondary structure predictions. The table *dataset_properties* contains the scientific name of the species, its taxonomy, the species’ abbreviation, and the release version of the protein annotation dataset.

The predicted MXE candidates are stored in the *exons* table. Each exon record is linked to a protein, and includes the 5'- and 3'-end positions of the exon with respect to the contig/chromosome, the exon number of the originally annotated exon, and the score and the length difference as parameters for the similarity of the predicted exon. An exon entry might contain annotations like an overlap with either an exon of another annotated isoform of the gene or with an exon of a neighbouring gene, and the mapping of transcriptome data (e.g. cDNA data). In order to retain annotations with respect to the same genome target sequences in case that MXE predictions will be repeated with different parameters or based on new releases of protein annotations, target specific exon annotations like location specific comments, manually verified exon positions and manually entered *trans*-spliced exons are stored in independent database tables.

### The web interface

As web application framework we chose Ruby on Rails since it has the advantage of rapid and agile development while keeping the code well organized. The site makes extensive use of Ajax (Asynchronous JavaScript and XML) in order to present the user a feature rich interface while minimizing the amount of transferred data. All technologies used are freely available and open source. The system is running on a Linux machine.

### Search options

**Figure 1:**
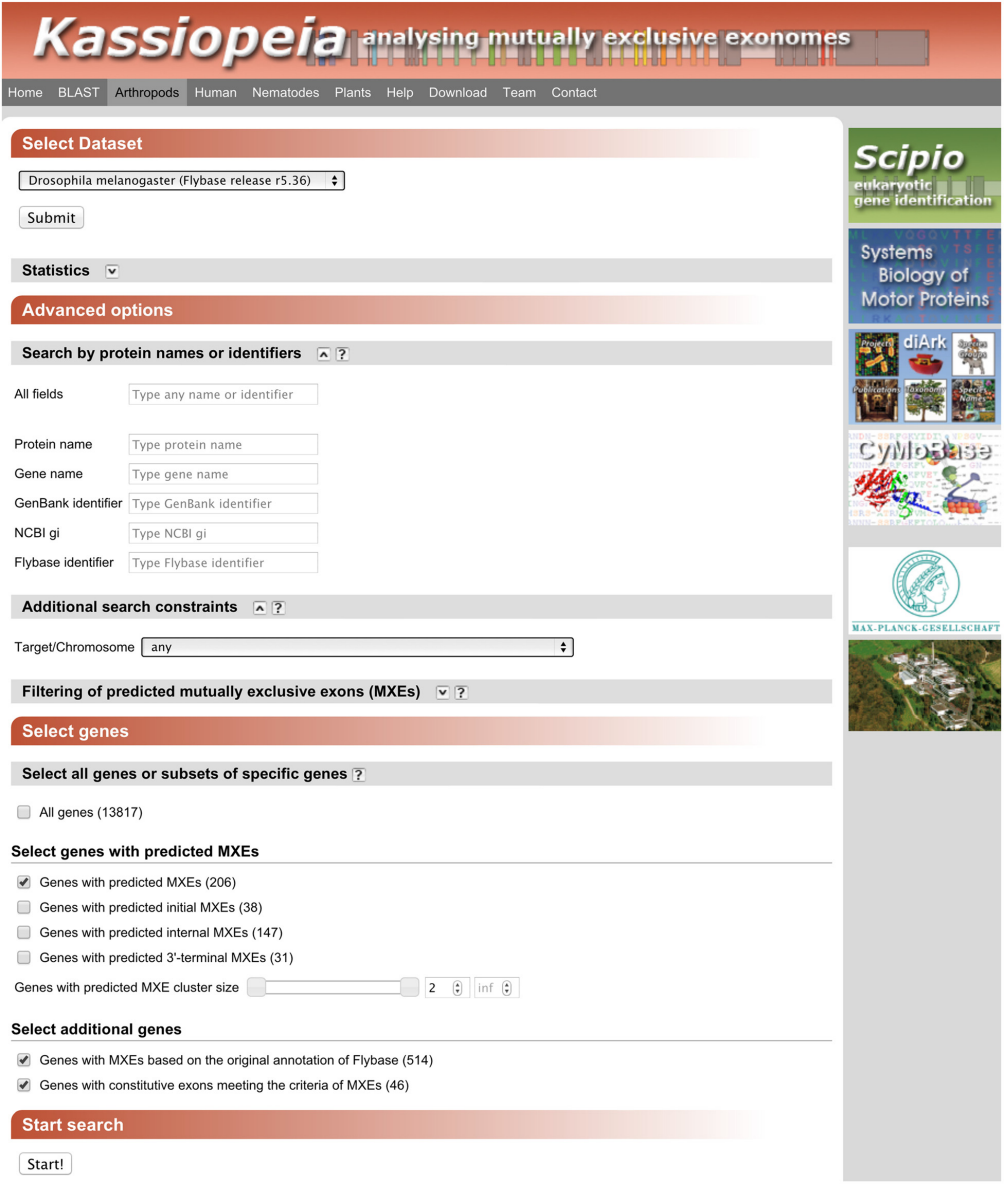
**Dataset selection and search options.** The Kassiopeia web application provides an interface to select a dataset from various species and taxa, to search for specific gene names and identifiers, and to choose a specific set of genes. In the example, the *D. melanogaster* dataset was selected comprising more than 13,000 genes, of which more than 200 contain predicted MXE candidates.

The web interface has been designed to provide easy access to the data while providing specific search and filter options for the expert (Figure 1). A BLAST [22] service provides a homology-based search against all datasets. The BLAST results are linked to gene-specific pages for further inspection. The entry to genome-wide analyses is via taxon-specific pages. Here, datasets corresponding to the available species can be chosen (Figure 1, top). Each dataset can be searched by protein name, gene name, and further identifiers as used in other databases (Figure 1, middle). Autocomplete widgets provide suggestions for matching names. In addition, single targets can be selected to restrict the analysis to, for example, a specific chromosome. From the filtered gene dataset, single or combined subsets can subsequently be selected, which are either all genes, genes for which MXE candidates were predicted, genes which were annotated in Flybase/Phytozome/Wormbase/NCBI as mutually exclusive spliced, and/or genes containing neighbouring exons annotated as constitutive or cassette exons, which match the criteria for MXEs using default values (Figure 1, bottom). Cassette exons are differentially included exons. If these are neighboured to exons with similar sequence, splice sites and reading frame (MXE criteria), MXE candidates will be predicted for the isoforms lacking the cassette exons. These MXE candidates represent false positive predictions or indicate false annotations, which can only be distinguished with the help of experimental data.

### Exon filtering

The default values (minimal socre of 15% and maximal length difference of 20 residues) for the MXE prediction parameters are reliable to reproduce most of the existing annotations. Applying these values to whole genome MXE predictions already results in many new MXE candidates. Relaxing these values will result in both the identification of more divergent MXE candidates as well as the prediction of false MXE candidates, which can only be distinguished by manual inspection and analysis of the respective cases. In order not to force users to repeat searches with less stringent values, we used relaxed values in the Kassiopeia prediction pipeline (see below). The user can then freely adjust the default values for all prediction parameters to more restricted or relaxed values within the advanced options in the Kassiopeia web interface.

**Figure 2:**
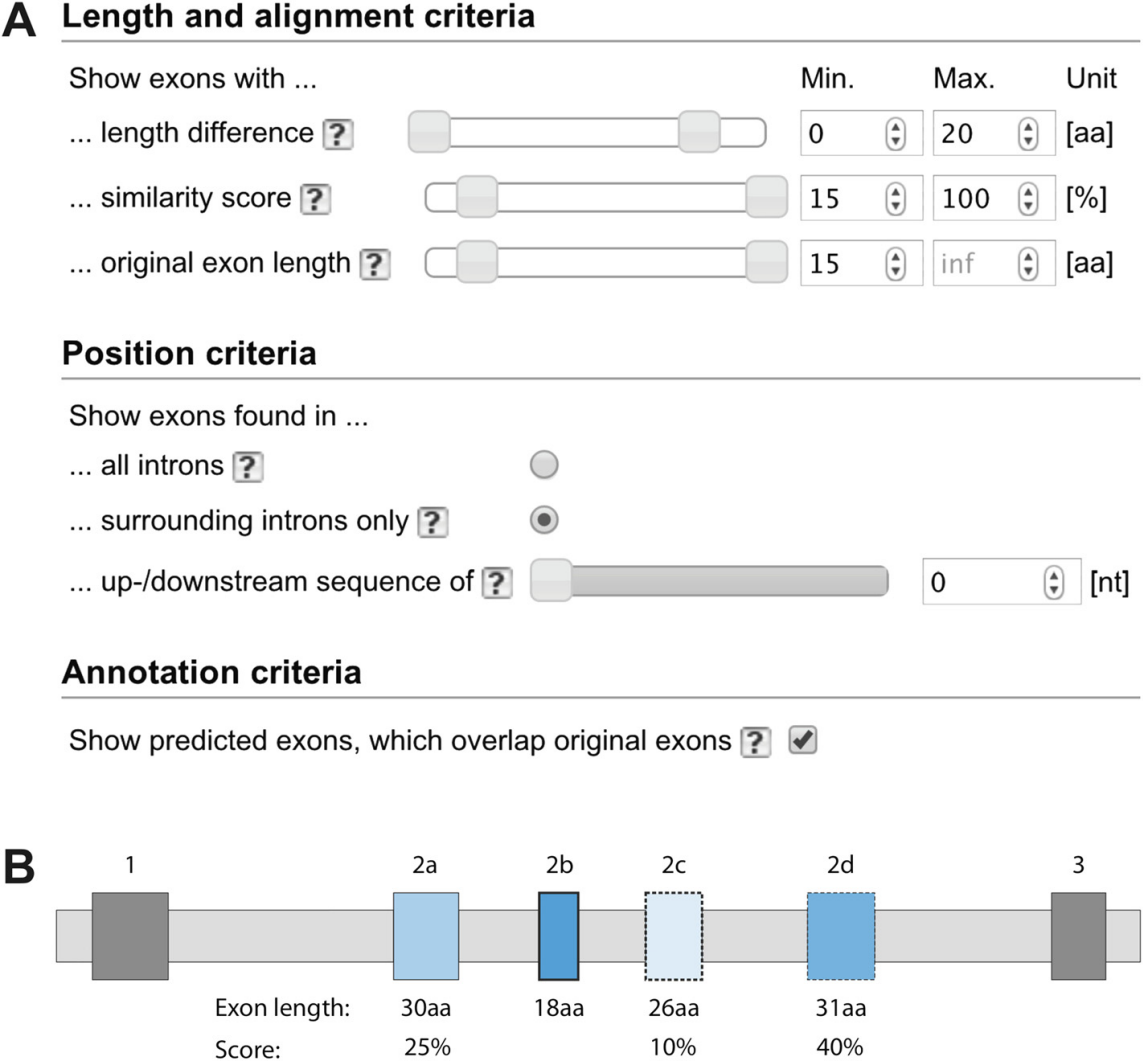
**Exon filtering. A)** Within Kassiopeia predicted MXE candidates can be filtered by the parameters of the MXE search algorithm and a filter to exclude predicted exons, which overlap with exons of other transcripts or genes. **B)** The effects of the different filter parameters are demonstrated on the example of a hypothetical gene containing a cluster of four MXEs. The gene includes three exons in its original annotation, exons 1, 2b, and 3 (constitutive exons are displayed as dark grey boxes; light-gray boxes denote introns). The algorithm found alternative exon 2a 5’ of 2b, and the two alternative exons 2c and 2d in the intron between exons 2b and 3. The exon candidates of the cluster of MXEs are drawn in blue. Scores and lengths of the predicted exons are given to demonstrate the potential effect of the filters. Dashed borderlines around MXEs indicate predicted exons that are not present in any annotated isoform, in contrast to continuous lines that indicate exons already annotated in at least one isoform. Exons with a thick borderline were manually verified by EST data, cross-species gene data, or have already been described in the literature.

The consequences of changing the default values for filtering the predicted MXEs (Figure 2A) will be explained on the example of a hypothetical cluster of four MXEs as shown in Figure 2B. In this example the original annotation contained the exons 1, 2b, and 3. For exon 2b one alternative exon was predicted in the 5’ intron between exons 1 and 2b (exon 2a) and two alternative exons were found in the 3’ intron (exons 2c and 2d). If the maximal allowed length difference between the original annotated exon (exon 2b) and the predicted exons (exons 2a, 2c, and 2d) were changed to less than 12 amino acids, exons 2a and 2d would be filtered out. The similarity score for MXEs is given in percent and defined by the alignment score of the amino acid sequence coded by the original exon to the one of the predicted exon divided by the alignment score of the amino acid sequence coded by the original exon to itself. Given the default minimal score of 15%, exon 2c in the example would be filtered out (Figure 2B). The minimal original exon length filter allows preventing predictions based on very short exons. If the minimal exon length were set to a value higher than 18 amino acids, all MXE candidates would be filtered out (Figure 2B).

According to our criteria, MXEs are expected to be located next to each other as part of a cluster. Because annotations might contain mis-predicted exons within a cluster of MXEs the Kassiopeia prediction pipeline was set up to search for exon candidates in all introns. By default, only those MXEs are selected that were predicted in the introns surrounding the original exon (Figure 2A). To allow the identification of MXE candidates in those partial genes, in which the 5’- and/or 3’-ends of the genes are missing, the exon prediction has been extended into the up- and downstream regions of the genes. The length of these regions, for which predicted MXE candidates are displayed, can be varied. However, this option must be treated with caution, because the number of false positive predictions might increase. Cases for false positives are clusters of terminal exons, whose inclusion in the transcripts is regulated by multiple promoters or multiple poly(A) sites and not at the level of splicing, and exons from tandem gene duplicates and *trans*-spliced genes [16]. Copies of several exons in the up- or downstream regions with the same order as in the original gene indicate gene duplicates and *trans*-spliced genes. Although not directly related to MXEs, these potentially *trans*-spliced genes and tandem gene duplicates can be displayed by selecting predicted exons found in all introns.

If the original annotations contain several isoforms of a gene, predicted exons in one isoform might overlap with exons of another isoform. If these predicted exons overlap but do not exactly match to an exon in another isoform of the original annotation they are potentially false positive predictions and can be deselected (Figure 2A).

### View options and statistics

In the view options section of the results the width of the exons in the graphical output can be scaled and some statistics based on the search results are provided.

### Graphical output and download options

**Figure 3:**
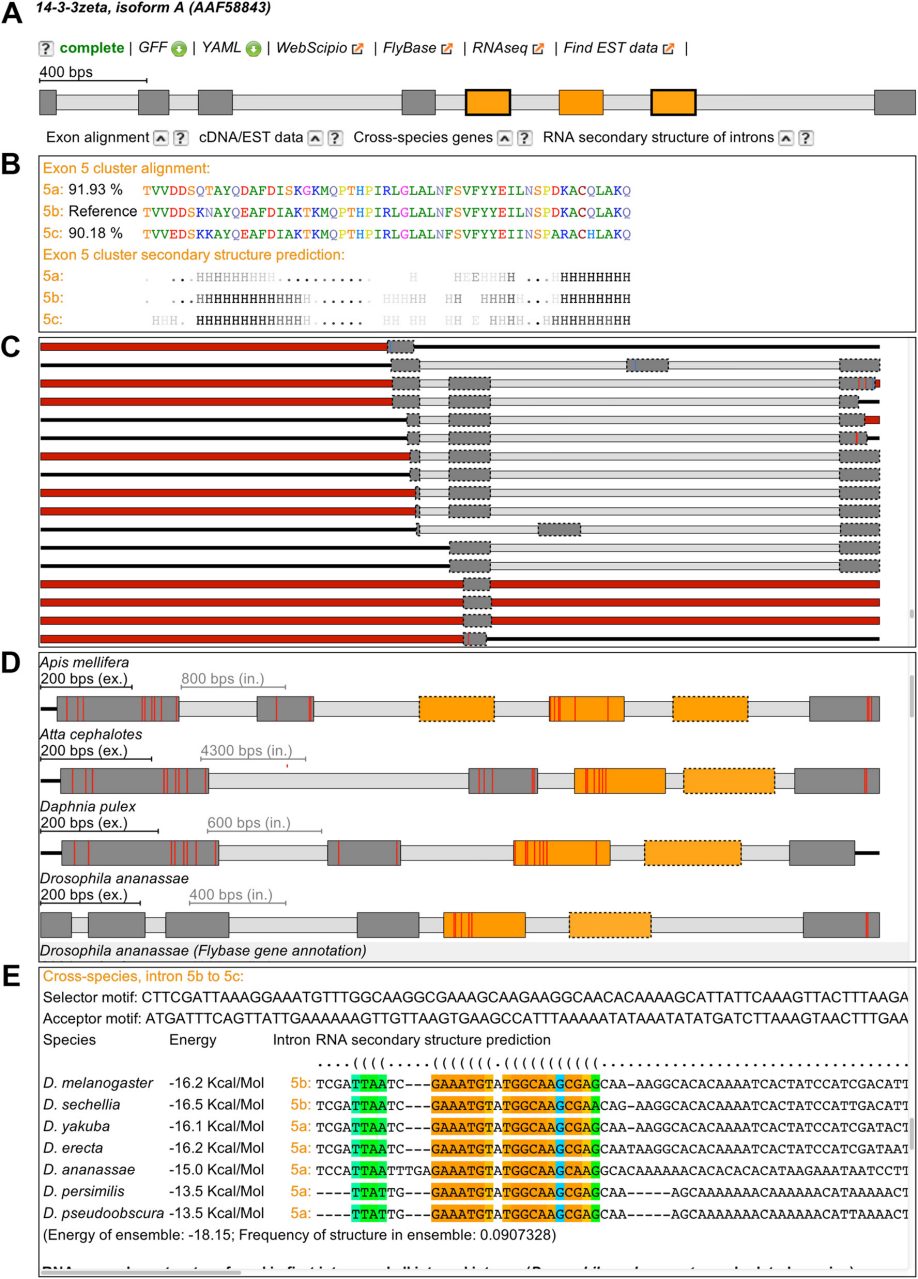
**The *Drosophila melanogaster 14-3-3ζ *gene as available in Kassiopeia.** The scheme of the exon-intron structure contains exons as dark gray boxes and introns as light gray boxes **(A)**. Exons of a cluster of MXEs have the same colour. The opacity of the predicted exons indicates the similarity to the original exon. The sequence alignments and secondary structure predictions **(B)**, additional evidence by EST data mapping **(C)**, cross-species search results **(D)**, and RNA secondary structure predictions **(E)** can be opened below the gene structure scheme.

The search results are shown as lists of genes represented by the exon-intron structures (Figure 3A). The gene structure schemes are generated and displayed in the Scalable Vector Graphics (SVG) format for resolution-independent scaling and for convenient interaction with specific graphical elements using JavaScript. For gene colouring we adopted the system used in WebScipio [21]. Exons in a cluster of MXEs get the same colour and the opacity denotes the similarity to the original search exon. Dashed lines around exons indicate newly predicted MXEs and continuous lines mark exons that have already been annotated as MXEs in Flybase/Phytozome/Wormbase/NCBI (Figure 2B). Thick lines indicate exons that were verified as MXEs by manually inspecting matching EST data, cross-species search results or literature mining. Constitutive exons with a thick green border represent exons that match our criteria of MXEs based on the default values. If several isoforms for one gene were present in the annotation datasets, an additional exon-intron structure picture would be shown for each isoform. Above the gene structure schemes, a label indicates the completeness or incompleteness of the exon-intron structure. Complete denotes genes for which all amino acids of the protein sequence from the annotation dataset could be mapped onto the genomic sequence. Incomplete gene structures contain gaps (protein sequence not found in the target genome), mismatches and/or sequence shifts. Details of the gene structures can be analysed by clicking on the WebScipio link on top of the gene structure picture. Below the gene structure schemes, sequence alignments and secondary structure comparisons of the MXE candidates are shown (Figure 3B) and, if available, additional evidence for the MXEs. The alignments of the amino acid sequences encoded by the exons in the cluster were generated with MUSCLE [23,24] and the secondary structure predictions were done with PSIPRED [25]. The gene structure schemes of the genes and isoforms can be downloaded directly and via WebScipio, which provides visual access to all details of the gene structure (e.g. in the alignment view every single exon and its corresponding translation can be inspected including intron splice sites and problems in the genome assembly) and many possibilities to download specific and global data for further processing (exons, introns, genomic DNA, transcript sequence, translation, etc.). In addition, pre-computed datasets of MXE containing genes and all MXE candidates are provided, although these are restricted to the data obtained with default values.

### Data resources and the MXE prediction pipeline

For the prediction of MXEs, annotations for 12 *Drosophila* species, for *Arabidopsis thaliana*, for *Caenorhabditis elegans* and for *Homo sapiens* were obtained from Flybase, Phytozome, Wormbase and NCBI, respectively:

– ftp://ftp.flybase.net/genomes:dmel_r5.36_FB2011_04, dana_r1.3_FB2011_07, dere_r1.3_FB2011_08, dgri_r1.3_FB2010_02, dmoj_r1.3_FB2011_05, dper_r1.3_FB2010_02, dpse_r2.25_FB2011_10, dsec_r1.3_FB2011_08, dsim_r1.3_FB2011_08, dvir_r1.2_FB2011_07, dwil_r1.3_FB2010_02, dyak_r1.3_FB2011_08

– ftp://ftp.arabidopsis.org/home/tair/Genes: TAIR10_genome_release

– ftp://ftp.wormbase.org/pub/wormbase/releases: WS230

– ftp://ftp.ncbi.nih.gov/genomes/H_sapiens: Build 37.3

To standardize the procedure for the predictions a pipeline was developed and run for each organism. The pipeline was designed as general as possible to incorporate any annotated genome sequence in the future. As input the pipeline requires the genome sequence and the annotated protein sequences, both in FASTA format. During the prediction process several scripts are started, which were written in the Ruby programming language and C/C++. Within Ruby we use BioRuby [26] to handle the sequences. The outputs of the prediction pipeline are YAML files.

### Reconstruction of gene structures

The first step in the prediction process is the generation of the exome of each organism by mapping the protein sequences onto the genomes using Scipio [27]. Scipio is able to recognize and report shifts in the reading frames of translated genomic sequences, mismatches between the protein query sequence and the translation of the genome sequence, questionable introns that do not match the prevalent intron splice site patterns GT---AG or GC---AG, and missing stop codons (Additional file 1). In some cases small parts of the protein sequences could not be identified in the gene regions due to mis-assembled regions or gaps in the genome sequence resulting in gaps in the reconstructed genes. These data are missed in the predictions but are, however, insignificant. For example, 64 out of 13,817 reconstructed genes in *D. melanogaster* contain a gap (0.46%; Additional file 1). Gene reconstructions that include these sequence-mapping problems are marked as incomplete in the results section of Kassiopeia.

### Prediction of mutually exclusive spliced exons

**Figure 4:**
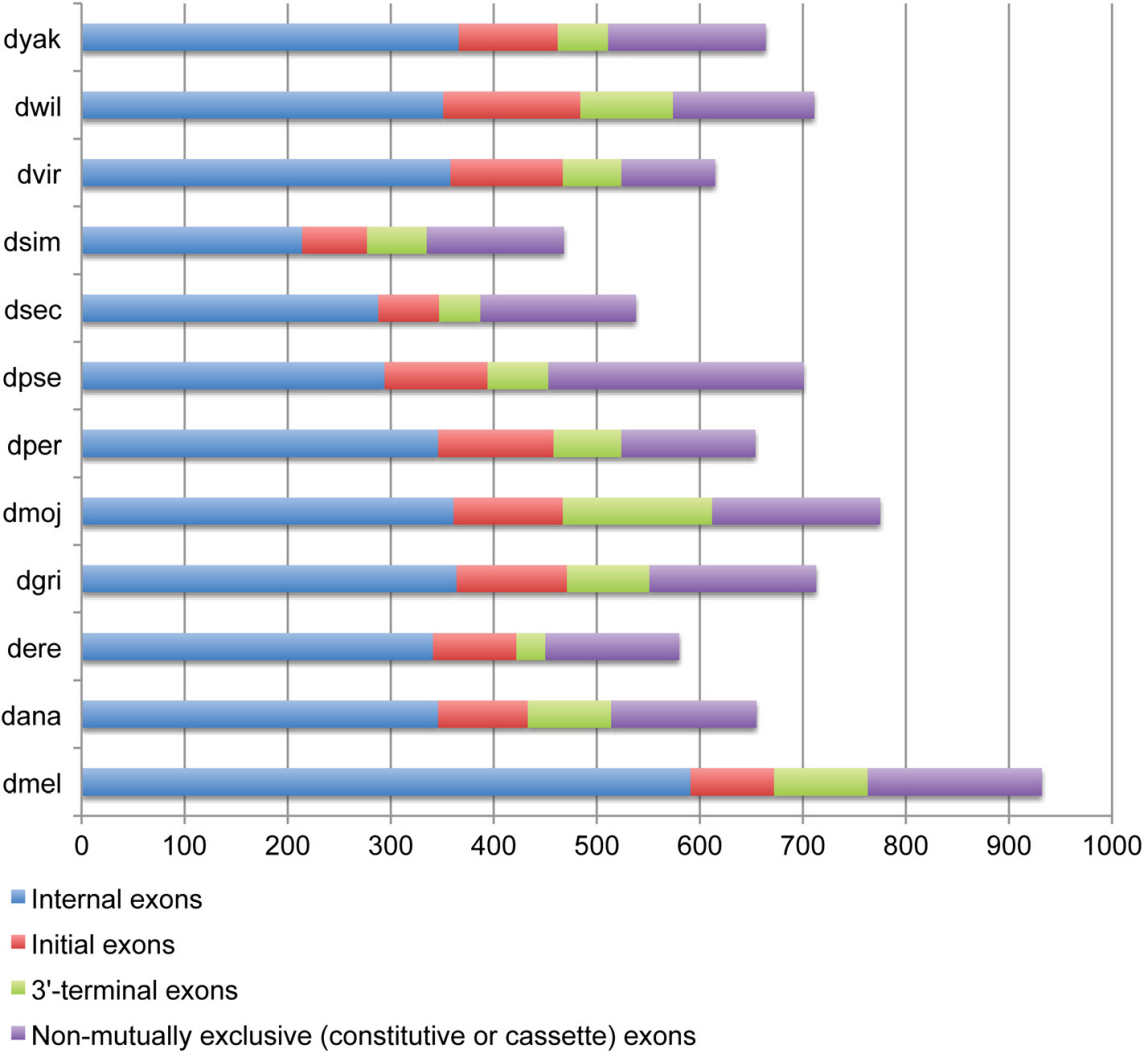
**Exons in the *Drosophila *genomes that appear in clusters of exons with same reading frames, splice sites, similar lengths and sequence similarity.** The coloured bars indicate the numbers of predicted internal exons, initial exons and 3’-terminal exons. The exons denoted as non-mutually exclusive match the criteria of MXEs, but have been annotated as constitutive or cassette exons.

**Figure 5:**
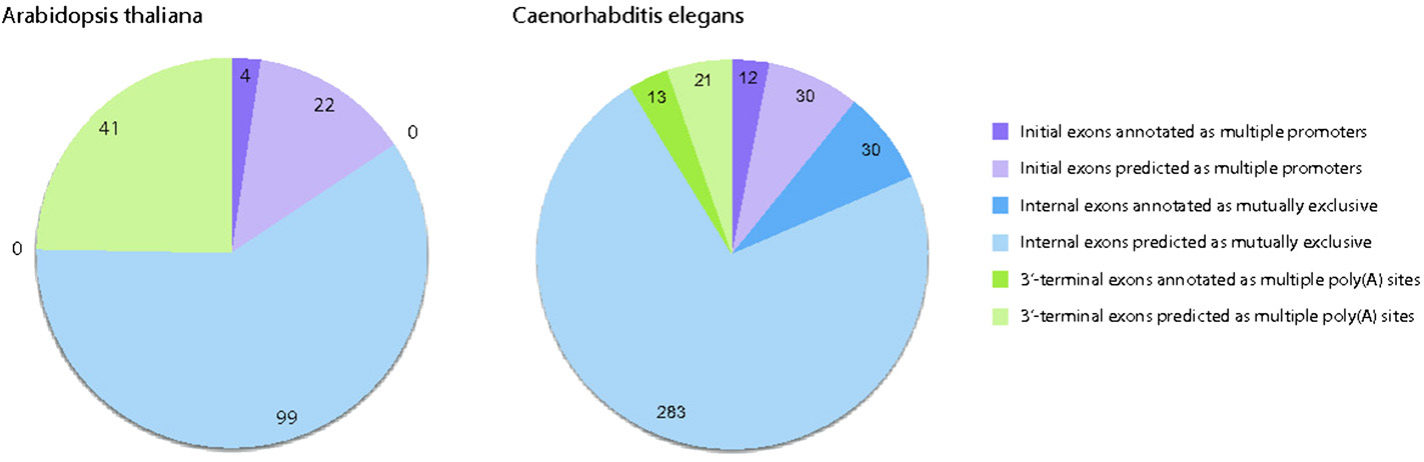
**Exons in the *Arabidopsis thaliana *and *Caenorhabditis elegans *genomes annotated and predicted as mutually exclusive exons.** The graphs represent the number of predicted initial, internal and 3’-terminal exons. Some of these predicted exons were already included in the annotations from Phytozome and Wormbase. The initial exons are supposed to be spliced by the multiple promoters mechanism and the 3’-terminal exons by the multiple poly(A) site mechanism.

MXEs were predicted in each reconstructed gene using the algorithm described in [16]. If a gene codes for several isoforms, the predictions were done independently for each isoform. The values for the parameters of the prediction pipeline were chosen to be slightly less stringent than the default values of Webscipio, which were used in the analyses. This means that more distantly related exon candidates, being true MXEs or potentially false positive predictions, were predicted during the process and are stored in Kassiopeia. The intention was to allow the user to apply appropriate filters to balance the amount of false positive and false negative predictions during the analysis without having to repeat the overall prediction. In the prediction pipeline the following values were used: a maximal length difference of 20 amino acids, a minimal score of 10%, and a minimal original exon length of 10 amino acids. MXE candidates were predicted in all introns and in 20,000 nucleotides up- and downstream of the respective gene. The analyses shown here (Table 1, Figures 4 and 5) are based on the default values of the MXE search of WebScipio, which are the following: a maximal length difference of 20 amino acids, a minimal score of 15%, a minimal original exon lengh of 15 amino acids, and exons are predicted in surrounding introns only and not in the up- and downstream regions. The default values are rather strict and more distantly related exons might be missed.

**Table 1:** **Statistics of the mutually exclusive exomes of 12 *Drosophila *** **species**

**Species**	**dmel**	**dana**	**dere**	**dgri**	**dmoj**	**dper**	**dpse**	**dsec**	**dsim**	**dvir**	**dwil**	**dyak**
Genes	13817	14917	14842	14635	14431	16639	15805	15936	15261	14353	15359	15845
Proteins	23554	15067	15046	14982	14590	16858	16594	16460	15353	14488	15507	16074
Genes with …												
… multiple exons	11054	11760	11541	11464	11214	12693	11952	12251	11798	11267	11549	12262
… predicted MXEs	206	153	134	168	181	178	171	127	137	166	191	167
… MXEs based on the original annotation	514	0	0	0	0	0	0	0	0	0	0	0
… constitutive exons sharing the criteria of MXEs	46	95	75	87	87	77	93	79	69	51	86	87
Exons in original annotation	60401	55971	55563	55602	54355	58060	57671	57240	52756	54441	55934	57989
Predicted MXEs	775	514	450	551	612	524	453	387	335	524	574	511
MXEs based on the original annotation	1297	0	0	0	0	0	0	0	0	0	0	0
Constitutive exons sharing the criteria of MXEs	169	141	130	162	163	130	248	151	133	91	137	153

### Additional evidence for mutually exclusive spliced exons

Experimental validation for the MXEs can be obtained from Expressed Sequence Tags (EST), cDNA and RNA-seq data. Therefore, we mapped EST data onto the respective gene regions and list hits below the gene structure schemes (Figure 3C). EST data for these comparisons were retrieved from the EST database of NCBI. The mapping was done by an extension to WebScipio [28].

Further confidence for the predicted MXEs can be obtained from similar searches in the homologous genes of related organisms. Thus we used Scipio’s cross-species search option [21] to identify and reconstruct orthologous genes in related species (Figure 3D). These genes were then used as basis for the prediction of MXEs. Here, default values were used for the prediction, except that MXEs were searched not only in the surrounding introns of the exons but also in all introns. These predictions are therefore independent of the ones in the original species.

Recently, it has been shown that mutually exclusive splicing can be directed by competing intron RNA secondary structures, which was first observed in *Drosophila*[20,29-31], but might also exist in mammalian species [32]. Although such competing RNA secondary structures have not yet been found in all clusters of MXEs [30,31], their identification would provide strong further confidence to any prediction. Therefore, we implemented a software pipeline to predict sites in the introns, which could build RNA secondary structures to regulate splicing (Figure 3E). The binding windows were computed using a genetic programming algorithm [29]. The first step in this process is the identification of binding windows within the intron preceding the cluster and the internal introns of the cluster, and within the internal introns and the intron following the cluster. Binding windows were predicted for all candidate clusters of MXEs using the SeqAn [33] and the ViennaRNA [34] packages, and, subsequently, also for the available exon-intron gene structures from the related species as obtained in the cross-species searches. For the latter, the identified binding windows of all homologous genes from the different species were aligned using MUSCLE [23,24] and the RNA secondary structures predicted by RNAalifold [35] from the ViennaRNA package. These analyses to add confidence to the predicted exon candidates were performed for all twelve *Drosophila* datasets and the *A. thaliana* dataset. Although several analyses have shown that RNA secondary structure predictions, which are based on comparative sequence analyses of coding or non-coding RNAs, are highly accurate (see for example [36]), these predictions are less accurate for RNAs obtained from intron DNA. The accuracy of the predictions can considerably be improved by thorough multi-species intron alignments, which are, however, difficult to generate automatically and reliably.

## Utility and discussion

Here, we present the web application Kassiopeia that allows exploring the content of MXEs in whole genomes. Currently, MXE candidate predictions for twelve *Drosophila* genomes, the *Arabidopsis thaliana* genome, the *Caenorhabditis elegans* genome and the human genome are available. Only the *Drosophila* data has already been analysed in detail [19], so that the other data has to be regarded as preliminary. A pipeline for the standardized prediction of MXE candidates has been implemented. The main part of the pipeline is the algorithm for the prediction of MXEs, which is implemented in WebScipio [16]. The predictions were compared with annotations as available from the respective species databases. Further evidence for predicted exons was obtained *in silico* through validation with EST data, comparison with predictions in orthologous genes of related species, and RNA secondary structure predictions. Kassiopeia allows homology-based searching, and selecting and filtering specific parts of the data. Thus, the user can browse the data for specific genes as well as for lists of candidates depending on the prediction parameters. Kassiopeia has been designed to easily adopt the data of any further analysed species, and the data from upcoming versions of genome annotations without loosing the results from the validations and annotations.

### Mutually exclusive exome data for twelve Drosophila species

The exomes of twelve completely sequenced *Drosophila* species [37], *D. melanogaster* (dmel), *D. ananassae* (dana), *D. erecta* (dere), *D. grimshawi* (dgri), *D. mojavensis* (dmoj), *D. persimilis* (dper), *D. pseudoobscura* (dpse), *D. sechellia* (dsec), *D. simulans* (dsim), *D. virilis* (dvir), *D. willistoni* (dwil), and *D. yakuba* (dyak), were reconstructed to subsequently predict exons that are spliced in a mutually exclusive manner. The annotations from Flybase contain between 13,817 and 16,639 genes for each species (Table 1 and Additional file 1). Alternative splice forms are well annotated in *D. melanogaster* (23,554 protein isoforms), but almost absent in the datasets of the other *Drosophila* species. The *Drosophila* species contain 52,756 to 60,401 annotated exons. 335 to 775 exons were predicted to be candidates for MXEs (Table 1). In the *D. melanogaster* genome 1,297 exons of the 60,401 exons were already annotated as MXEs (Table 1). Here, MXEs were defined as being annotated if the exons are in a cluster of neighbouring exons and each of the annotated isoforms of the corresponding gene includes exactly and only one of the exons of the cluster independently of the position of the cluster within the gene. However, most of these exons are terminal exons, which are alternatively included in the transcripts in conjunction with the use of alternative transcription initiation or 3’-end processing sites, whose regulation need not be at the level of splicing [38]. Of the 1,297 annotated MXEs in *D. melanogaster* only 261 are internal exons, whose splicing is supposed to be regulated by the formation of specific RNA secondary structures [30,31]. Using WebScipio's default values, 218 exons out of these 261 exons can be reconstructed resulting in a sensitivity of 83.5% [19]. Figure 4 displays the number of predicted MXEs of all twelve *Drosophila* species divided into three types: initial 5’-end exons, internal exons, and 3’-terminal exons. In addition, the number of exons that have been annotated as constitutive or cassette exons but match the criteria of MXEs are shown. In contrast to the sensitivity, we cannot determine a reliable estimate for the specificity, which considers the false positive predictions. Evaluating the specificity would require a perfectly annotated genome including the knowledge that specific introns, for which we predict MXEs, definitively do not contain any further exons. Future experiments providing further cDNA, EST and RNA-Seq data could help in determining the specificity by either confirming the predictions or by assigning the exons as constitutive or cassette types.

The annotations available for the other *Drosophila* species do not contain any annotated MXE (Table 1). Therefore, many of the potential MXEs have been annotated as constitutive exons. For example, all exons of the clusters of MXEs in the well-known *muscle myosin heavy chain*[17] and *DSCAM* genes [16,20] have been annotated as constitutive. We have already shown that many of the predicted MXEs of the *D. melanogaster* X chromosome were also identified as exons in an *ab initio* gene prediction with AUGUSTUS [16]. Therefore we suppose that most of the 129 exons in *D. melanogaster*, which were annotated as constitutive but are not supported by cDNA/EST data yet, might also constitute MXEs [19].

### Mutually exclusive exome data for *Arabidopsis thaliana* and *Caenorhabditis elegans*

*Arabidopsis thaliana* and *Caenorhabditis elegans* were chosen as representatives for plants and nematodes, respectively, because they are designated model species and many single gene studies as well as whole transcriptome analyses have been performed. Thus, their annotations are supposed to belong to the best available. In the *A. thaliana* genome 166 exons were predicted to be mutually exclusive spliced belonging to 66 genes. 26 of them are initial exons, which are supposed to be spliced by the multiple promoters mechanism, and 41 are 3’-terminal exons containing multiple poly(A) sites (Figure 5). Thus, 99 exons are candidates for MXEs. In TAIR (The Arabidopsis Information Resource) 139 exons are annotated as MXEs, of which only 14 are internal exons. Those exons are, however, of very different length passing WebScipios search algorithm. Of Kassiopeia's predicted MXE candidates only four initial exons but no internal or 3’-terminal exons were already annotated as mutually exclusive in the *A. thaliana* gene dataset (Figure 5). Our analysis provides the first evidence, that mutually exclusive splicing is also a widely used mechanism to increase the potential number of transcripts in plants. Within PubMed and ArabiTag, which is a database to a recent very comprehensive analysis of alternative splicing events in *A. thaliana*[39], mutually exclusive spliced genes in *A. thaliana* are not described at all.

In the *C. elegans* genome 389 exons were predicted to be mutually exclusive spliced belonging to 138 genes. 42 of them are initial exons, 313 are internal exons and 34 are 3’-terminal exons (Figure 5). In the case of *C. elegans* many of the predicted exons are already annotated in Wormbase: 12 initial exons, 30 internal exons, and 13 3’-terminal exons. However, apart from the terminal exons we identified 283 new candidates for MXEs in internal clusters, about five times more than the largest number of MXE candidates reported (55 exons; [40]). These examples show that with Kassiopeia it is possible to identify many new candidates for mutually exclusive spliced genes that were not covered by exhaustive EST data sequencing yet.

## Conclusions

Mutually exclusive splicing is a highly regulated mechanism leading to the inclusion of one exon of a cluster of neighbouring exons into the final transcript. We have set up a pipeline to predict MXE candidates in the whole genomes of several model organisms based on conserved splice sites, same reading frame, sequence similarity and similar length. To make these data easily accessible and informative, we constructed Kassiopeia, a web interface in which researchers can BLAST and search for specific proteins, or browse through whole genomes or chromosomes. For each gene Kassiopeia provides a comprehensive gene structure scheme, amino acid sequence alignments and predicted secondary structures of the MXEs, and, if available, further confidence to putative MXEs from cDNA/EST data, comparative predictions in closely related species, and RNA secondary structure predictions. As standard values for the search, Kassiopeia offers those with which MXEs in well-described genes like the *DSCAM* and the *muscle myosin heavy chain* gene could be reproduced. However, the user can adjust these values to search for more divergent exon candidates.

## Availability and requirements

Kassiopeia is maintained under the GPL license and can be accessed at http://www.motorprotein.de/kassiopeia.

## Competing interests

The authors declare that they have no competing interests.

## Authors’ contributions

KH and MK set the requirements for the system. KH wrote the software. KH and MK extensively tested the software, performed all analyses and wrote the manuscript. All authors read and approved the final version of the manuscript.

## Supplementary Material

Additional file 1 **Extensive statistics of the mutually exclusive exomes of 12 *Drosophila *** **species.** Click here for file
