# Supplementary material for: Kassiopeia: a database and web application for the analysis of mutually exclusive exomes of eukaryotes
Source: BMC Genomics. 2014 Feb 10;15:115. doi: 10.1186/1471-2164-15-115 (PMC3923563; doi:10.1186/1471-2164-15-115)
Supplement: Additional file 1 — Extensive statistics of the mutually exclusive exomes of 12 Drosophila species. [file 1471-2164-15-115-S1.pdf]

Additional file 1. Extensive statistics of the mutually exclusive exomes of 12 *Drosophila* species. Species abbreviations are *D. melanogaster* (dmel), *D. ananassae* (dana), *D. erecta* (dere), *D. grimshawi* (dgri), *D. mojavensis* (dmoj), *D. persimilis* (dper), *D. pseudoobscura* (dpse), *D. sechellia* (dsec), *D. simulans* (dsim), *D. virilis* (dvir), *D. willistoni* (dwil), and *D. yakuba* (dyak).

Species	dmel	dana	dere	dgri	dmoj	dper	dpse	dsec	dsim	dvir	dwil	dyak
Genes	13817	14917	14842	14635	14431	16639	15805	15936	15261	14353	15359	15845
Genes with …												
… multiple exons	11054	11760	11541	11464	11214	12693	11952	12251	11798	11267	11549	12262
… predicted MXEs	206	153	134	168	181	178	171	127	137	166	191	167
… MXEs based on the original annotation	514	0	0	0	0	0	0	0	0	0	0	0
… constitutive exons sharing the criteria of MXEs	46	95	75	87	87	77	93	79	69	51	86	87
… gap	64	117	102	109	107	282	122	204	202	111	99	129
... sequence shift	19	34	28	31	37	170	31	105	96	27	30	41
… mismatches	46	37	35	48	42	65	50	55	45	41	45	47
… questionable intron	39	136	126	162	158	311	125	229	278	130	176	157
… missing stopcodon	63	10	2	4	2	10	22	9	7	4	11	5
Proteins	23554	15067	15046	14982	14590	16858	16594	16460	15353	14488	15507	16074
Proteins with …												
… multiple exons	19986	11795	11602	11596	11275	12775	12645	12415	11816	11294	11610	12411
… predicted MXEs	804	153	134	169	181	180	203	128	137	166	192	168
… MXEs based on the original annotation	2255	0	0	0	0	0	0	0	0	0	0	0
… constitutive exons sharing the criteria of MXEs	147	96	75	88	87	77	107	79	69	51	86	87
… gap	137	117	102	109	107	282	129	204	202	111	99	131
... sequence shift	33	34	28	36	37	173	31	105	96	27	31	41
… mismatches	67	37	35	48	42	66	53	56	45	41	46	48
… questionable intron	83	147	127	163	158	311	128	235	278	130	176	160
… missing stopcodon	77	10	2	4	2	10	23	9	7	4	12	5
Exons in original annotation (no duplicates)	60401	55971	55563	55602	54355	58060	57671	57240	52756	54441	55934	57989
Predicted MXEs (no duplicates)	775	514	450	551	612	524	453	387	335	524	574	511
MXEs based on the original annotation (no duplicates)	1297	0	0	0	0	0	0	0	0	0	0	0
Constitutive exons sharing the criteria of MXEs (no duplicates)	169	141	130	162	163	130	248	151	133	91	137	153
